# A Topological Description of Hubs in Amino Acid Interaction Networks

**DOI:** 10.1155/2010/257512

**Published:** 2010-05-26

**Authors:** Omar Gaci

**Affiliations:** Le Havre University, LITIS EA 4108, BP 540, 76058 Le Havre, France

## Abstract

We represent proteins by amino acid interaction networks. This is a graph whose vertices are the proteins amino acids and whose edges are the interactions between them. Once we have compared this type of graphs to the general model of scale-free networks, we analyze the existence of nodes which highly interact, the hubs. We describe these nodes taking into account their position in the primary structure to study their apparition frequency in the folded proteins. Finally, we observe that their interaction level is a consequence of the general rules which govern the folding process.

## 1. Introduction

Proteins are biological macromolecules participating in the large majority of processes which govern organisms. The roles played by proteins are varied and complex. Certain proteins, called enzymes, act as catalysts and increase several orders of magnitude, with a remarkable specificity, and the speed of multiple chemical reactions essential to the organism survival. Proteins are also used for storage and transport of small molecules or ions, control the passage of molecules through the cell membranes, and so forth. Hormones, which transmit information and allow the regulation of complex cellular processes, are also proteins.

Genome sequencing projects generate an ever increasing number of protein sequences. For example, the Human Genome Project has identified over 30,000 genes [3] which may encode about 100,000 proteins. One of the first tasks when annotating a new genome is to assign functions to the proteins produced by the genes. To fully understand the biological functions of proteins, the knowledge of their structure is essential.

In their natural environment, proteins adopt a native compact three-dimensional form. This process is called folding and is not fully understood. The process is a result of interactions between the protein's amino acids which form chemical bonds.

In this study, we treat proteins as networks of interacting amino acid pairs [4]. In particular, we consider the subgraph induced by the set of amino acids participating in the secondary structure also called Secondary Structure Elements (SSE). We call this graph SSE interaction network (SSE-IN). We carry out a study to identify the main properties that the SSE-INs share with the scale-free model. Studying the degree distributions, we are interested in the existence of hubs which are nodes whose degree is big. Then, we describe these specific nodes by their neighbourhood in the folded proteins to find a correlation with their hydrophobicity or their position in the primary structure.

In [5–8] the authors compare also similar models of amino acid interaction networks to the scale-free model to describe some of their properties. In particular, the authors want to study the node degree distribution, the inner coreor outer layer mean degrees. All those studies are led in order to identify the general topological properties of amino acid interaction networks. Here, we do the same since we want to identify the topological criteria which imply that a node acts as a hub.

### 1.1. Amino Acid Interaction Networks

The 3D structure of a protein is represented by the coordinates of its atoms. This information is available in Protein Data Bank (PDB) [9], which regroups all experimentally solved protein structures. Using the coordinates of two atoms, one can compute the distance between them. We define the distance between two amino acids as the distance between their *C*_*α*_ atoms. Considering the *C*_*α*_ atom as a “center” of the amino acid is an approximation, but it works well enough for our purposes. Let us denote by *N* the number of amino acids in the protein. A contact map matrix is an *N* × *N* 0-1 matrix, whose element (*i*, *j*) is one if there is a contact between amino acids *i* and *j* and zero otherwise. It provides useful information about the protein. For example, the secondary structure elements can be identified using this matrix. Indeed, *α*-helices spread along the main diagonal, while *β*-sheets appear as bands parallel or perpendicular to the main diagonal [10]. There are different ways to define the contact between two amino acids. Our notion is based on spacial proximity, so that the contact map can consider noncovalent interactions. We say that two amino acids are in contact iff the distance between them is below a given threshold. A commonly used threshold is 7 Å [11] and this is the value we use.

**Figure 1:**
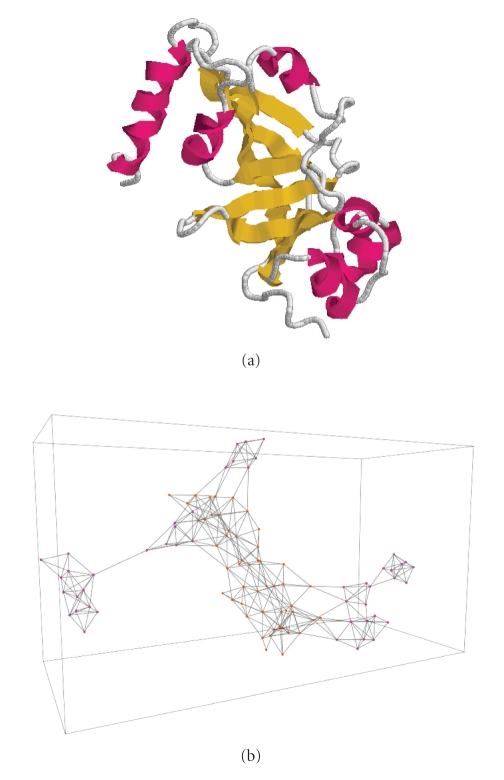
Protein 1DTP (a) and its SSE-IN (b). From a pdb file, a parser we have developed produces a new file which corresponds to the SSE-IN graph displayed by the GraphStream library [1].

Consider a graph with *N* vertices (each vertex corresponds to an amino acid) and the contact map matrix as incidence matrix. It is called contact map graph. The contact map graph is an abstract description of the protein structure taking into account only the interactions between the amino acids. Now let us consider the subgraph induced by the set of amino acids participating in SSE. We call this graph SSE interaction network (SSE-IN) and this is the object we study in the present paper. The reason of ignoring the amino acids not participating in SSE is simple. Evolution tends to preserve the structural core of proteins composed from SSE. On the other hand, the loops (regions between SSE) are not so important to the structure and hence, are subject to more mutations. That is why homologous proteins tend to have relatively preserved structural cores and variable loop regions. Thus, the structure determining interactions are those between amino acids belonging to the same SSE on local level and between different SSEs on global level. Figure 1 gives an example of a protein and its SSE-IN.

## 2. The Scale-Free Network

The most important property of scale-free systems is their invariance to changes in scale. The term scale-free refers to a system defined by a functional form *f*(*x*) that remains unchanged within a multiplicative factor under rescaling of the independent variable *x*. Indeed, this means power-law forms, since these are the only solutions to *f*(*a* *n*) = *b* *f*(*n*), where *n* is the number of vertices [12]. The scale-invariance property means that any part of the scale-free network is stochastically similar to the whole network and parameters are assumed to be independent of the system size [13].

One of the most important network properties is the degree distribution of vertices. A degree of a vertex is the number of edges incident to it. The mean degree of a network is the mean of the degrees of all vertices. For a network with *n* vertices and *m* edges, the mean degree is *z* = 2*m*/*n*. We will note by *p*_*k*_ the ratio of vertices having degree *k* (or the probability that a vertex has a degree *k*). The values *p*_*k*_ define the degree distribution of a network. The cumulative degree distribution *P*_*k*_ = ∑_*i*=*k*_^*∞*^ *p*_*k*_ is the probability for a vertex to have a degree at least *k*.

**Figure 2:**
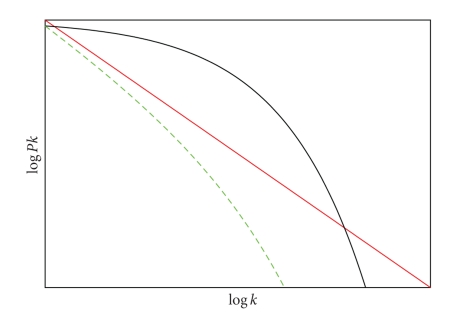
Degree distribution for each the three models described by Amaral [2]. The red line follows a power law, a function with a relatively “fat tail” as for scale-free networks. The green line corresponds to truncated scale-free networks because it describes a power law regime followed by a sharp cut-off. The black curve has a fast decaying tail, typically exponential, and corresponds to single-scale networks.

The random graphs of Erdõs and Rényi [14, 15] are the most studied network model. They have Poisson degree distribution. However, many real networks have different degree distributions. Amaral et al. [2] have studied networks that can be classified into three groups according to the shape of their cumulative degree distribution; see Figure 2. First, scale-free networks are those with power law distribution *p*_*k*_ ~ *k* ^−*α*^ or *P*_*k*_ ~ *k* ^−(*α*−1)^, a function which decreases polynomially with *k*. The second class is single scale networks with exponential degree distribution *P*_*k*_ ~ *e* ^−*k*/*α*^. This distribution decreases exponentially, much faster than the previous. The third class is broad-scale or truncated scale-free networks with distribution.

(1) $$\begin{array}{c} P_{k}\sim k^{-(\alpha -1)}e^{-k/\alpha }. \end{array}$$

This distribution is somewhere between the previous two, a power law regime followed by a sharp exponential cutoff. The common feature of these classes is that most of the vertices have low degree and there exist a small number of high degree nodes. The last are called hubs and play an important role for the connectivity of the whole network.

## 3. Experimental Results

### 3.1. Previous Works

In [16] we have studied the degree distribution of amino acid interaction networks. We have shown that the SSE-INs have a cumulative degree distribution which can be approximated by the function *P*_*k*_:

(2) $$\begin{array}{c} P_{k}=1.48347k^{0.962515}{\exp\;}^{-k/2.12615}. \end{array}$$

Thus, protein SSE-INs are truncated scale-free networks; this is also confirmed by previous studies [7, 8, 17].

**Figure 3:**
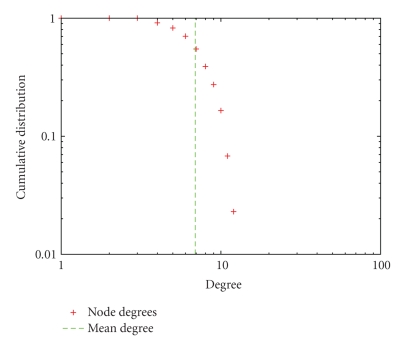
Cumulative degree distribution for 1COY SSE-IN. The curve decreases quickly for degrees superior to the mean degree *z* which acts as a threshold.

As well, we have shown that the mean degree values constitute a threshold for protein SSE-IN cumulative degree distribution. For degrees lower than *z*, the cumulative distribution decreases slowly and after this threshold its decrease is fast compared to an exponential one; see Figure 3.

In the present paper, we continue to describe SSE-INs by comparing them to the scale-free model. Since the SSE-INs are truncated scale-free networks, we are interested in the identification of hubs. We begin our study by defining a hub, and then we want to show that there exist specific criteria which ensure that a node is a hub. Thus, we will present specific topological measures form SSE-INs which describe how a node acts as a hub.

The dataset we use is the same than the one exploited in [16]; see Table 1. We have selected proteins relying on the SCOP v1.73 classification and notably the fold families. Our dataset is composed of proteins from families belonging to the main four classes; they count more than 100 proteins. We have selected a total of 18296 proteins. We use a broad sample of proteins to guaranty more general results and avoid fluctuations.

### 3.2. Hubs Identification

**Figure 4:**
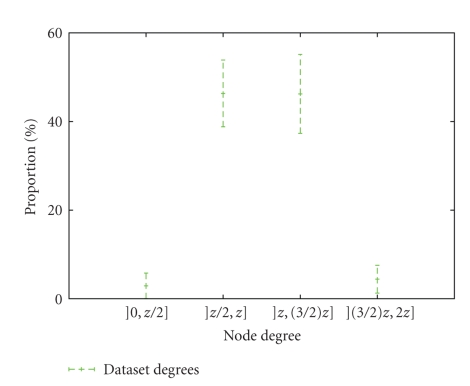
Degree of nodes in all studied SSE-IN as a function their mean degree *z*. For each studied SSE-IN, we compare the degree of each node to the SSE-IN mean degree *z*. Less than 5% of nodes are hubs; they have a degree superior to (3/2)*z*.

In [16], we have shown that the degree distributions depend on the mean degree values. Then, we compare for each node its degree to the mean degree denoted *z* (see Figure 4) to illustrate how nodes interact and in particular to highlight the weak fraction of highly connected nodes, also called hubs. Further, we consider a hub as a node whose degree *k*_hub_ satisfies: *k*_hub_ > (3/2)*z*. The hubs represent less than 5% of the total node number; see Figure 4.

An interesting study is to put in evidence the biological properties of nodes whose connectivity is marginal. Thus, we want to identify the hubs which highly interact with their neighbours in the folded proteins.

To this end, we have proceeded by grouping the proteins according to their secondary structures. Indeed, we have already shown [18] that the protein SSE-IN topologies from structural classifications are homogeneous and established a parallel between structural and topological properties. Based on the SCOP classification and more precisely on the fold families, we have selected a total of 18296 proteins, see Table 1, and studied their SSE-IN to describe the hub specificities.

**Figure 5:**
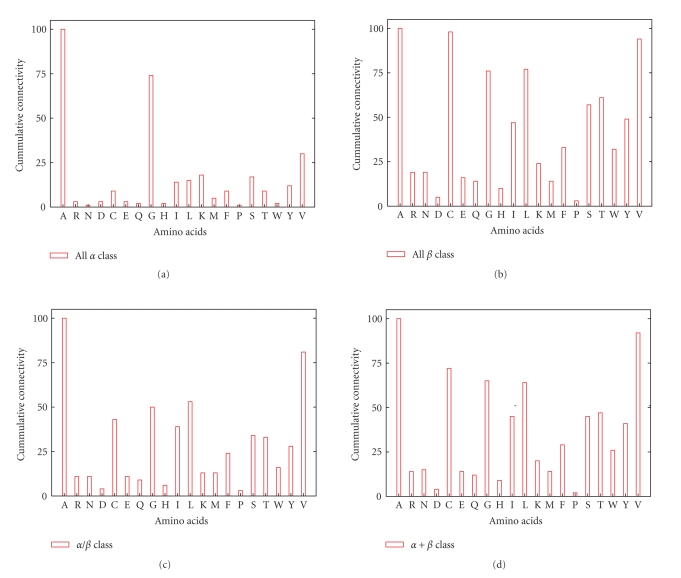
Each time a hub appears in an SSE-IN, we sum its degree according to the amino acid it represents. We repeat the process grouping the SSE-IN by their family and also by their structural class. We normalization to obtain the cumulative connectivity by class. The amino acid Ala acts more often as hub independently of the protein classifications.

For each protein SSE-IN belonging to the same SCOP structural family, we identify the nodes which are hubs; see Figure 4. Then, we group the hubs according to the amino acids they represent and sum their degrees to obtain the amino acid connectivity score by fold families. By repeating this process at the SCOP class level, we calculate and normalize the accumulated connectivity level of amino acids playing the role of hubs; see Figure 5.

If we consider that the role played by an amino acid inside a folded protein is equivalent to its interaction degree, then these plots show that despite a functional diversity between the four SCOP classes, there are globally the same amino acids which interact most, namely, the Ala, Cys, Gly, Leu, Val. Therefore, the amino acids having a high connectivity interact independently of the protein biological function.

From a biological viewpoint, these observations illustrate perfectly the general folded protein shape that is, the hydrophobic side chains are packed into the interior of the protein creating a hydrophobic core and a hydrophilic surface. Then, the first reason for which certain amino acids interact most is correlated with their hydrophobicity.

**Figure 6:**
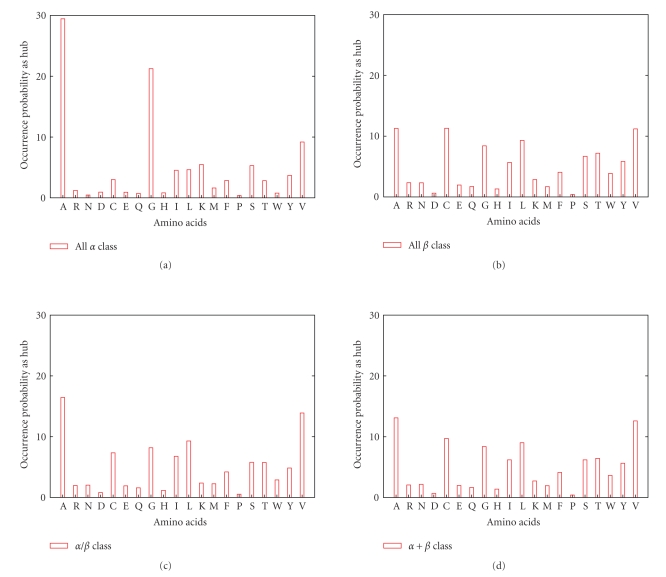
Each time a hub interacts, we add one to its occurrence score and then deduce an occurrence probability for the amino acids to act as a hub in an SSE-IN. We repeat the process grouping the SSE-INs by their family and also by their structural class. The probability of an amino acid Ala being a hub is the stronger independently of the protein classifications.

We also compute the occurrence level of hubs, that is their number of appearances on each protein SSE-IN. We accumulated this score at the SCOP class level to obtain the probability for each amino acid to be a hub in a protein SSE-IN; see Figure 6. We can remark the existence of peaks which show clearly a strong tendency of amino acids Ala, Cys, Gly, Leu, and Val to have a high interactivity within the folded protein. Thus, there are the same amino acids which play the role of hubs independently of structural families.

**Figure 7:**
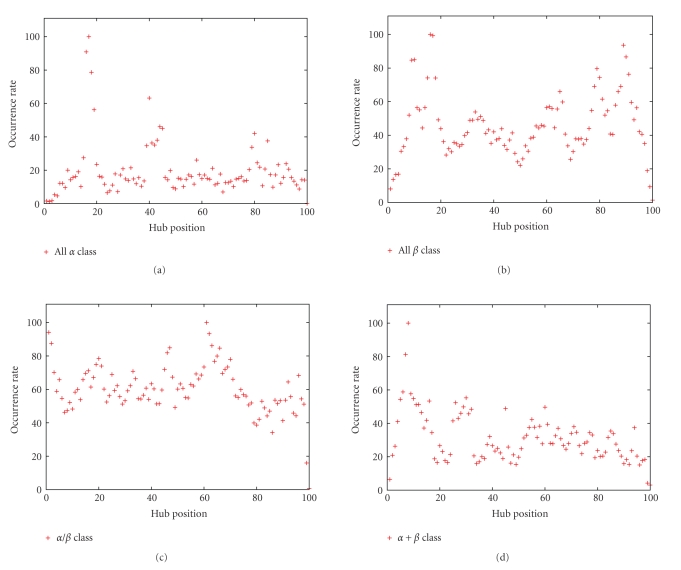
We assign to each node a number so that the H extremity has the number 100. We sum the occurrence score of hubs according to their positions and normalize. We observe favorable regions when the occurrence rate is high.

Now, we want to describe the way in which the hubs appear in the folded protein. Then, we study the distribution of hub positions as a function of the SSE-IN structural class to identify variations dependent or not on the biological function of proteins. To lead this study, we attribute to the nodes an incremental position so that the H extremity has a position 100. Then, each time a hub exists in an SSE-IN, we increment its occurrence number by position and finally normalize by the maximum to obtain the occurrence ratio of hubs according to their positions in the SSE-IN; see Figure 7. The results show the existence of favorable regions in which the hub apparition is higher than somewhere else. This favorable localization is strongly visible for the *All* *α* class where the hubs have a tendency to interact around the positions 20, 40, or 80. The distribution of hub positions is the most homogeneous for the *α*/*β* class. It involves dependence on the SSE-IN topology since it is not possible to find more than one strong favorable area which appears around the position 60.

To illustrate the existence of hubs favorable localization in the SSE-IN, we rely on the rich-club phenomenon [19] according to which the hubs have a tendency to be connected to one another. We compute the rich-club connectivity of a hub as the ratio of the number of links to the maximum number of links between nodes belonging to the rich-club.

**Figure 8:**
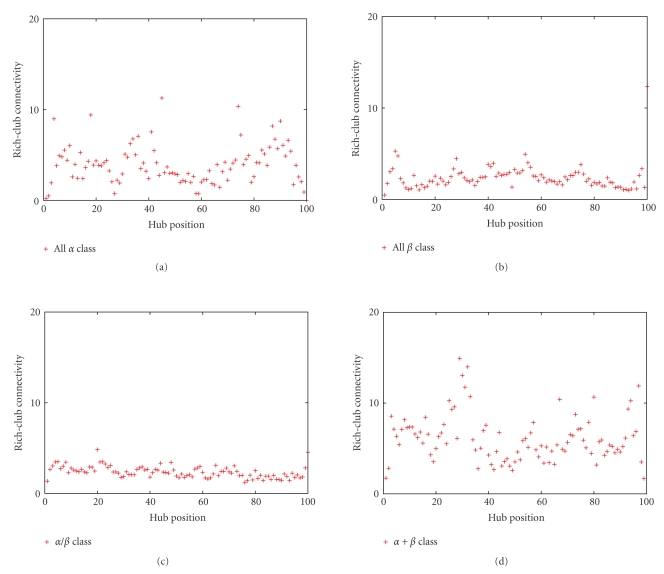
Rich-club connectivity according to the hub positions. When this measure is high, the hub neighbourhood is composed in majority of other hubs.

It appears, see Figure 8, that certain hubs are isolated, mainly when the rich-club connectivity is low (positions 0, 30, and 60 for the *All* *α* class) whereas the favorable hub localizations correspond to a high coefficient.

The main observations about hubs behavior are the following. First, there are five amino acids which have a stronger probability to have a high connectivity. Second, the hubs have a tendency to act in particular region in the SSE-IN.

**Figure 9:**
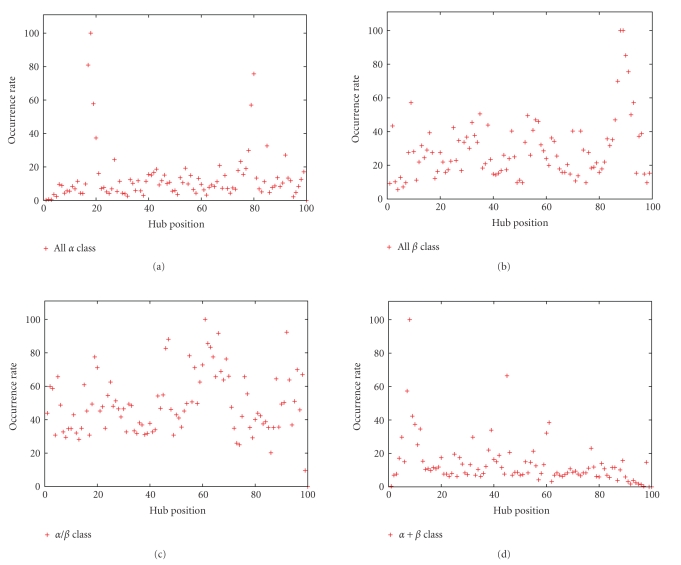
Occurrence rate of the hub Ala for each SCOP class level. Comparing these plots to Figure 7, it appears that the amino acid Ala corresponds to the higher occurrence rate.

These two observations lead us to compute only the occurrence rate of the most frequently encountered hub Ala according to its position; see Figure 9. By comparing the Figures 7 and 9, it appears clearly that the highest occurrence rate for the four classes corresponds to the position of the amino acid Ala. Therefore, we can establish a relation between the amino acid position in the primary structure and the hub apparition. Thus, the amino acids Ala, Cys, Gly, Leu, and Val act as hub because they are localized in the protein sequence in favorable regions which involve a high interaction within the folded structure. This tendency is actually a consequence of the amino acid hydrophobicity and more globally it is a consequence of the formation of a hydrophobic core in the folded proteins.

## 4. Conclusion

In this paper, we study the nodes whose connectivity is marginal in amino acid interaction network. We want to understand what the topological conditions which ensure that a node acts as a hub are. The study of hubs shows that there exist amino acids which play a central role independently of the protein biological functions. The degree of hubs depends before all on their hydrophobicity and is also a consequence of the node position in the protein sequence.

The study we present shows mainly that the amino acid interaction networks are graphs whose topological properties depend on the general folding rules.

The characterization we propose constitutes a first step of a new approach to the protein folding problem. The properties identified here, but also other properties we studied previosly, can give us an insight on the folding process. They can be used to guide a folding simulation in the topological pathway from unfolded to folded state.

## Figures and Tables

**Table 1:** Structural families studied for the scale-free properties. We choose only families which count more than 100 proteins, for a total of 18296 proteins. We select a protein only when all its domains are the same. We have worked with the SCOP v1.73 classification.

Class	Number of families	Number of proteins
*A* *l* *l* *α*	12	2970
*A* *l* *l* *β*	17	6372
*α*/*β*	18	5197
*α* + *β*	16	3757
